# *Leishmania amazonensis* DNA in wild females of *Lutzomyia cruzi* (Diptera: Psychodidae) in the state of Mato Grosso do Sul, Brazil

**DOI:** 10.1590/0074-02760150317

**Published:** 2015-12

**Authors:** Everton Falcão de Oliveira, Aline Etelvina Casaril, Nathália Lopes Fontoura Mateus, Paula Guerra Murat, Wagner Souza Fernandes, Elisa Teruya Oshiro, Alessandra Gutierrez de Oliveira, Eunice Aparecida Bianchi Galati

**Affiliations:** 1 Universidade de São Paulo, Faculdade de Saúde Pública, Programa de Pós-Graduação em Saúde Pública, São Paulo, SP, Brasil; 2 Universidade Federal de Mato Grosso do Sul, Centro de Ciências Biológicas e da Saúde, Campo Grande, MS, Brasil; 3 Universidade de São Paulo, Faculdade de Saúde Pública, Departamento de Epidemiologia, São Paulo, SP, Brasil

**Keywords:** natural infection, Lutzomyia cruzi, Leishmania amazonensis, molecular biology, polymerase chain reaction, dissection

## Abstract

Studies on natural infection by *Leishmania* spp of sandflies collected in endemic and nonendemic areas can provide important information on the distribution and intensity of the transmission of these parasites. This study sought to investigate the natural infection by *Leishmania*in wild female sandflies. The specimens were caught in the city of Corumbá, state of Mato Grosso do Sul (Brazil) between October 2012-March 2014, and dissected to investigate flagellates and/or submitted to molecular analysis to detect *Leishmania* DNA. A total of 1,164 females (77.56% of which were *Lutzomyia cruzi*) representing 11 species were investigated using molecular analysis; 126 specimens of *Lu. cruzi*were dissected and also submitted to molecular analysis. The infection rate based on the presence of *Leishmania* DNA considering all the sandfly species analysed was 0.69%; only *Leishmania (Leishmania) amazonensis* was identified in *Lu. cruzi* by the molecular analysis. The dissections were negative for flagellates. This is the first record of the presence of *L. (L.) amazonensis* DNA in *Lu. cruzi*, and the first record of this parasite in this area. These findings point to the need for further investigation into the possible role of this sandfly as vector of this parasite.

The urbanisation of the human population and the transformation of the eminently rural cycle of leishmaniasis into a concomitantly urban and periurban phenomenon with the adaptation of some species of sandflies to urban environments have contributed to an increase in the incidence of the disease in Brazil in recent decades (Gontijo & Melo 2004, Lainson & Rangel 2005, Tauil 2006). According to the Information System on Notifiable Diseases (SINAN), 3,245 cases of visceral leishmaniasis (VL) and 3,188 cases of cutaneous leishmaniasis (CL) in humans were confirmed in the state of Mato Grosso do Sul (MS) between January 1999-December 2014 (dtr2004.saude.gov.br/sinanweb). In the city of Corumbá (MS), one of the oldest urban VL foci registered in Brazil, 104 human cases of VL and 21 of CL were confirmed between 2001-2013 (dtr2004.saude.gov.br/sinanweb). However, there are no studies on genotyping of *Leishmania* species in this city, where*Lutzomyia cruzi* (Mangabeira, 1938) has been suspected as a vector of *Leishmania* (*Leishmania*) *infantum*[senior syn. of *Leishmania* (*Leishmania*)*infantum chagasi* (Cunha & Chagas, 1937)]. This suspicion was based on the absence of *Lutzomyia longipalpis* (Lutz & Neiva, 1912), the main vector of this parasite in the Americas, together with ecological and epidemiological evidence (Galati et al. 1997), the observation of flagellates in dissected females and their identification as *L. (L.) infantum* by monoclonal antibodies (Santos et al. 1998), and the detection of the kDNA of this parasite followed by hybridisation (Pita-Pereira et al. 2008). Additionally, the vectorial competence of *Lu. cruzi* for*L*. *(L.)infantum* and *Leishmania(Leishmania) amazonensis* Lainson & Shaw, 1972 was demonstrated experimentally when this sandfly bite and transmitted these parasites to hamsters (Oliveira 2015). Thus, the participation of this sandfly in the transmission of *Leishmania* spp should be more investigated.

Studies investigating the natural infection of vector insects are useful to detect the intensity of the transmission of *Leishmania* Ross, 1903 and to understand the eco-epidemiology of leishmaniasis. Such studies are essential to local health authorities in their attempts to establish prevention measures and evaluate the effectiveness of programs aimed at controlling the transmission of leishmaniasis (Michalsky et al. 2002, Martín-Sánchez et al. 2006).

The high level of the sensitivity and specificity of molecular methods regardless of the number, stages of the life cycle and location of the parasites in the sandfly’s gut (Perez et al. 1994, Pita-Pereira et al. 2005) are important for a better understanding of the epidemiology of leishmaniasis and the vector capacity of different species (Aransay et al. 2000, Perruolo et al. 2006). The polymerase chain reaction (PCR), widely employed in the analysis of entomological samples from different geographic regions (Feliciangeli et al. 1994, da Silva & Grunewald 1999, Aransay et al. 2000, Paiva et al. 2007), offers considerable sensitivity and specificity in the detection and identification of *Leishmania* species (Schönian et al. 2003).

The aim of the present study was to investigate the natural infection by*Leishmania* in wild female sandflies caught in Corumbá through dissection to investigate flagellates and/or detection of the*Leishmania* DNA.

## MATERIALS AND METHODS

*Study area* - The specimens used in the present investigation were caught between October 2012-March 2014 in the urban perimeter of Corumbá (19º00’33”S 57º39’12”W; 118 m above sea level), which is located in the northeastern portion of MS (Central-West Brazil). The municipality has an area of 64,962.8 km^2^, which represents 18.19% of the total area of the state, and is located 415 km from the state capital (Campo Grande) in the Pantanal wetland region on the border with Bolivia.

Five collection sites (convenience sampling) were determined in neighbourhoods with records of human cases of VL in the year prior to the beginning of the study: four residential areas in the peripheral region and one in the commercial district of the city. Table I displays a brief description of the characteristics of each collection site.

**TABLE I:** General characteristics of sampling sites in the city of Corumbá, state of Mato Grosso do Sul, Brazil, April 2012-March 2014

Residence (neighbourhood)	Geographical location	Domesticated animals (n)
Centro	Central region of city; sampling site closest to Paraguay River (approximately 500 m)	Dogs (2) Chicken (1)
Cristo Redentor	Southeastern periphery of the city	Dog (1)
Maria Leite	Northeastern periphery of the city	Dogs (2) Chickens (15^*a*^) Geese (5) Ducks (3)
Nova Corumbá	Southern periphery of the city	Dogs (5) Chickens (4) Cats (3)
Popular Nova	Southeastern periphery of the city	Dog (1)

*a*: the number of chickens at this residence varied throughout the study, but was always greater than 15.

*Collections and acquisition of sandflies for molecular analysis* - Automatic light traps were installed weekly in the peridomicile area of the five residences selected. For the identification of females, the genitalia were dissected on slides containing a drop of saline solution, whereas males were clarified and mounted on slides in balsam. The identification of both sexes was performed as described by Galati (2014). Engorged females and those whose entire bodies were clarified for the identification of the species were not included in the study.

In the first six months of analysis (October 2012-March 2013), females were grouped in pools of up to 10 insects of the same species, location and collection date, and placed individually in 1.5 mL microtubes with isopropyl alcohol and stored at -20ºC for subsequent PCR. The remaining specimens were placed individually in 1.5 mL microtubes.

*Collections and acquisition of sandflies for dissection* - A sample of females was dissected to investigate the presence of flagellates in accordance with the method described by Johnson et al. (1963). The specimens were caught with an aspirator in a chicken coop (the same collection site in the neighbourhood of Maria Leite used for the light trap collection) between 07:00 pm-09:00 pm on three different days (1 day in September 2013, 1 in November 2013, and the last in December 2013). After exposing the gut and spermathecae of the females for the investigation of flagellates and species identification, respectively, the contents on the slide (gut, thorax, and head) were transferred to 1.5 mL microtubes with isopropyl alcohol and stored at -20ºC for subsequent PCR. The specimens negative in the direct exam for flagellates were grouped in pools of up to ten.

*PCR and restriction fragment length polymorphism analyses* - For DNA extraction, the specimens were ground with the aid of a plastic pestle in 1.5 mL tubes with 300 μL of 5% Chelex^®^ resin solution (Bio-Rad, USA). The solution was mixed in a vortex for 15 s, centrifuged at 13,000 rpm for 60 s and placed in a water bath at 80ºC for 30 min. The vortex and centrifugation procedures were repeated and the supernatant was removed and transferred to a different sterile Eppendorf tube. The extraction product was stored at -20ºC.

PCR was performed targeting a region of the internal transcribed spacer (ITS) of the*Leishmania* ribosomal gene (ITS1) with approximately 300 bp. Five microlitre of the sample, 12.5 μL of GoTaq^®^ Green Master Mix (Promega, USA), 5.5 μL of water, and 1 μL of each oligonucleotide [LITSR (5’-CTGGATCATTTTCCGATG-3’) and L5.8S (5’-TGATACCACTTATCGCACTT-3’)] were added for a final reaction volume of 25 μL (El Tai et al. 2000). The following manipulations were the amplification conditions in the thermal cycler (BIOER, China): 95ºC for 3 min followed by 35 cycles of 95ºC for 30 s, 53ºC for 30 s, and 72ºC for 1 min, with post-extension at 72ºC for 5 min. The negative controls were a reaction without DNA containing water and DNA from nonfed F_1_ females. The positive controls were DNA from *L*.*(L.) infantum* (MHOM/BR/1972/BH46) and *L*.*(L.)amazonensis* (IFLA/BR/1967/PH8) extracted from cultures.

The PCR products were viewed using electrophoresis with 1.5% agarose gel in 100 mL of Tris-borate-ethylenediamine tetraacetic acid (TBE) buffer stained with GelRed^TM^ (Biotium, USA). The electrophoretic run was performed at 100 V for 100 min in concentrated TBE buffer. Viewing of the bands was performed using ultraviolet light with a 300-nm filter.

The products from positive samples were submitted to *Hae*III restriction enzyme digestion (isolated from *Haemophilus aegyptius*), which cleaves fragments in segments that have the 5’....GG^▼^CC....3’ or 3’....CC_▲_GG....5’ sequence to identify the species of*Leishmania* (Schönian et al. 2003). One microlitre of 10x buffer, one unit of *Hae*III enzyme and 1 mg of DNA from the PCR were used and the volume was completed with 10 mL of ultrapure water. The sample was incubated in a water bath at 37ºC overnight. The material was then submitted to electrophoresis in 2% polyacrylamide gel with TBE buffer for 3 h.

*Ethics* - This study received the approval of the Animal Experimentation Ethical Committee of the Federal University of Mato Grosso do Sul (Brazil) under process 491/2013. The research group has a permanent license for the collection of zoological material issued by the Brazilian Institute of Environment and Renewable Natural Resources (SISBio 25952-1).

The field studies were carried out on private lands and the owners gave permission to conduct the collections and acquisition of sandflies in their peridomicile areas. Further, the field studies did not involve endangered or protected species.

## RESULTS

During the weekly collections with light traps between October 2012-March 2014, 9,759 specimens (8,278 males and 1,481 females) were collected, belonging to 13 species:*Brumptomyia brumpti* (2♀), *Evandromyia cortelezzii* (4♀), *Evandromyia aldafalcaoae* (3♂, 5♀),*Evandromyia corumbaensis* (41♂, 115♀), *Evandromyia sallesi* (6♂, 16♀), *Evandromyia walkeri* (4♀),*Lu. cruzi* (8,061♂, 1,147♀), *Lutzomyia forattinii*(125♂, 159♀), *Micropygomyia peresi* (33♂, 10♀),*Martinsmyia oliveirai* (8♂, 12♀), *Psathyromyia bigeniculata* (1♂, 2♀), *Sciopemyia sordellii* (4♀), and*Nyssomyia whitmani* (1♀). Among the total number of females collected, 1,038 were investigated for natural infection by*Leishmania* using only PCR. Another 126 females collected with an aspirator were dissected for the study of flagellates and subsequently analysed by the same method. Thus, 1,164 females were analysed by PCR (Table II).

**TABLE II:** Distribution of the sandfly females investigated for natural infection by *Leishmania* according to species and type of analysis

Species	n (%)	Analysis method	
		Individual (n)	Pool (number of pools)
*Brumptomyia brumpti*	1 (0.09)	1	0 (0)
*Evandromyia aldafalcaoae*	3 (0.26)	3	0 (0)
*Evandromyia cortelezzii*	3 (0.26)	3	0 (0)
*Evandromyia corumbaensis*	95 (8.16)	91	4 (2)
*Evandromyia sallesi*	11 (0.95)	11	0 (0)
*Evandromyia walkeri*	1 (0.09)	1	0 (0)
*Lutzomyia cruzi*	903^*a*^ (77.58)	569	334 (57)
*Lutzomyia forattinii*	133 (11.43)	133	0 (0)
*Micropygomyia peresi*	1 (0.09)	1	0 (0)
*Martinsmyia oliveirai*	10 (0.86)	10	0 (0)
*Sciopemyia sordellii*	3 (0.26)	3	0 (0)
Total	1,164 (100)	826	338 (59)

*a*: one hundred twenty-six of 903*Lu*. *cruzi* females were dissected for flagellate study.

Only eight of the 1,164 females (0.69%) investigated exhibited a DNA band characteristic of *Leishmania* (300 bp). All naturally infected females were *Lu. cruzi*, caught on the same night in a single trap installed in the Maria Leite neighbourhood in June 2013. On this occasion, 272 sandflies were captured, 248 of them being males and 22 females, all of*Lu*. *cruzi*, and one male each of *Lu. forattinii* and of *Mt*. *oliveirai*. Thus, the natural infection rate of *Lu. cruzi* was 0.89% (8/903) based on the presence of *Leishmania* DNA. The parasite identified in all the amplified products was *L. (L.) amazonensis* (200 bp and 140 bp) (Figure). Regarding the 126 females dissected for the study of flagellates, all were negative with both methods employed for the investigation of natural infection by *Leishmania*.

**Figure:**
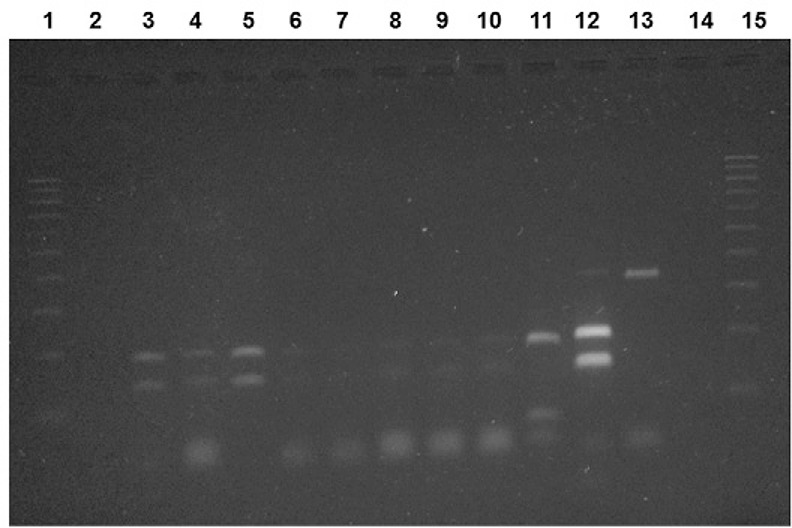
Digestion of amplified products from internal transcribed spacer 1 region of *Leishmania* with *Hae*III restriction enzyme. Lane 1: ladder marker with 100 bp; 2: negative control (reaction without DNA containing water); 3-10: sample of wild sandflies naturally infected by *Leishmania (Leishmania) amazonensis*; 11: positive control by *Leishmania (Leishmania)infantum* (MHOM/BR/1972/BH46); 12: positive control *L*. *(L.)amazonensis*(IFLA/BR/1967/PH8); 13: sample not digested by *Hae*III; 14: negative control (DNA from nonfed F1 females); 15: ladder marker of 100 bp.

## DISCUSSION

This study identified the natural infection of a sandfly species by a*Leishmania* species not yet reported during a regular survey of collections. Periodic surveys for the detection of natural infection by*Leishmania* are important for the understanding of the components of the parasite transmission chain. The detection of naturally infected sandfly species that have not previously been suspected as vectors of any*Leishmania* species demonstrates the need for studies to investigate their vectorial competence and the identification of permissive vectors (Kamhawi 2006, Paiva et al. 2007).

In the present study, the overall infection rate was 0.69% and the rate for*Lu. cruzi* alone was 0.89%. No flagellate forms were found in the dissected specimens and PCR was negative for all the pools analysed in this group. The females dissected were caught in a chicken coop close to the trap to which the positive females were attracted. In the chicken coop the females were collected with an aspirator while resting on the walls and birds. On the other hand, the light trap having light as its attraction may attract females which have had blood meals on various animals, including mammals which serve as reservoirs of*Leishmania.*

Although *Lu. cruzi* and *Lu. longipalpis* females are morphologically indistinguishable constituting a complex of species (Young & Duncan 1994, Galati 2014), and despite the fact that Santos et al. (2003) reported the collection of three*Lu. longipalpis* males in Corumbá, the finding of only*Lu. cruzi* males in the present investigation, as well as in previous studies (Galati et al. 1985, 19,^1997^, Santos et al. 1998, Pita-Pereira et al. 2008, Almeida et al. 2010, Casaril et al. 2014, Oliveira 2015), led us to identify all the females of this complex captured as *Lu. cruzi.*

Different methods with different degrees of sensitivity and specificity have been employed for the detection of natural infection in blood-feeding insects, such as dissection for the direct study of flagellates, inoculation in experimental animal models and isolation of the parasite in a culture medium with dissected insects (Deane 1956, Lainson et al. 1985, Sherlock 1996). Although more expensive in comparison with other methods, PCR is a practical tool with high degrees of sensitivity and specificity and allows the grouping of individuals in pools (Schönian et al. 2003, Paiva et al. 2006, Savani et al. 2009). However, pooling may lead to the underestimation of the natural infection rate, as it is not possible to identify how many individuals were actually infected. In such cases, the calculation of the minimum infection rate (Paiva et al. 2006, 2010) and the estimation of the prevalence of infection using an algorithm (Katholi et al. 1995, Martín-Sánchez et al. 2006) have been employed. In order to estimate more accurately the possible natural infection rate, it was decided to analyse the specimens caught in light traps during regular collections from April 2013 individually.

This is the first report of the natural infection of *Lu*.*cruzi* by *L*. *(L.)amazonensis*and of the presence of this parasite in Corumbá. Although *Lu*.*cruzi* has been studied little, the others reports of the finding of *Leishmania* DNA in wild females relate to*L.(L.)infantum*. Santos et al. (1998) found a 0.39% infection rate based on the dissection of 3,575 specimens of *Lu*. *cruzi*. Another 1,013 sandflies of seven different species were dissected and no flagellate forms were found. Based on these findings, the authors implicated *Lu*. *cruzi*as a vector of *L.(L.)infantum* in Corumbá (Santos et al. 1998). In the same municipality, Pita-Pereira et al. (2008) found a 1.5% minimum infection rate in *Lu*. *cruzi* by *L*.*(L.)infantum* using the minicircle region of kDNA as the target of multiplex PCR with hybridisation. The minicircle region of kDNA has a high degree of sensitivity and is capable of detecting minimal quantities of*Leishmania* DNA (Smyth et al. 1992, Freitas-Lidani 2014). However, the minicircle region of kDNA only permits the identification of the genus of the parasite (Schönian et al. 2003).

For *Lu.longipalpis*, which is a confirmed vector of *L. (L.)infantum*, the first record of wild females naturally infected by*L*. *(L.)amazonensis* occurred in the city of Antônio João, located in the southeastern portion of MS, on the border with Paraguay (Paiva et al. 2006). Subsequently, other authors also found *Lu*. *longipalpis* naturally infected by the same parasite in the city of Bonito (Savani et al. 2009). These two locations, Antônio João and Bonito, are respectively endemic for canine VL and CL.

The fact that the eight *Lu. cruzi* females were caught on a single night with a single CDC trap installed in a peridomicile area demonstrates that the parasite seems not to be dispersed throughout the urban area. Additionally, in this case, a common source is strongly suggested for natural infection because only nonengorged females were analysed. The observation of a rodent of the genus*Dasyprocta*, considered a secondary host of *L*.*(L.)amazonensis* (Lainson et al. 1994, Ashford 2000, Lainson & Shaw 2005), in the peridomicile area where the infected specimens were collected may explain these results. Factors that can contribute to the presence of this rodent in the area include the location of the dwelling on the outskirts of the town, a yard fenced with barbed wire, the presence of fruit trees, and a chicken coop, the base of which was suspended approximately 20 cm above the ground, leaving a clearance in which the animal was observed.

*L*. *(L.)amazonensis* has been recorded in Bolivia, Brazil, Colombia, French Guyana and Paraguay. In Brazil, this parasite has been found in all regions, especially the Amazon Region. However, it is likely that the geographical distribution of *L*. *(L.)amazonensis* is broader than is currently known and that it also extends into other countries of South America where its sandfly vector is found (Lainson & Shaw 1987, 2005,Grimaldi et al. 1989, MS/SVE 2010). This parasite is implicated as an etiological agent of CL (Lainson & Shaw 1987, 20, 2005). However, there are also human cases of VL, diffuse or anergic leishmaniasis and post-kala-azar dermal leishmaniasis attributed to this parasite (Barral et al. 1991, Aleixo et al. 2006) as well as canine VL (Tolezano et al. 2007,Hoffmann et al. 2012).

In the first year of study (2012), two autochthonous cases of CL were reported in the municipality of Corumbá. In the two following years (2013-2014), there were no reported cases of CL. Between January-July 2015, only one case of mucosal leishmaniasis was reported (MS 2015). However, due to lack of studies on aetiology and/or genotyping of*Leishmania* species in Corumbá, it is not possible infer that the aetiology of human cases of the disease.

As observed for *Lu*. *longipalpis*, it is possible that *Lu*. *cruzi* also has a permissive character and permits infection by other species of the genus *Leishmania*, which suggests that the adhesion mechanism of the parasite through lipophosphoglycans is not species specific or may occur by means of other mechanisms (Volf & Myskova 2007). This has important implications for the transmission and evolution of the parasite, as it may contribute to the dispersal of *Leishmania* due to its ability to adapt to new vectors (Myskova et al. 2007), since the main vector of *L*. *(L.)amazonensis,Bichromomyia flaviscutellata* (Mangabeira, 1942), has not yet been found in the region (Galati et al. 1985, 19, 1997, Santos et al. 1998, Almeida et al. 2010,Casaril et al. 2014, Oliveira 2015).

The incrimination and subsequent confirmation of a species as a vector of*Leishmania* should be based on several criteria, the first of which is the discovery of naturally infected wild females through the detection of the flagellate forms of the parasite on more than one occasion (Killick-Kendrick & Ward 1981, Killick-Kendrick 1990). Another criterion is the isolation and typing of promastigotes from females that have not fed for more than 36 h (Ready 2013).

Due to its epidemiological complexity, cutaneous and VL, the aetiology of which is attributed to *L*. *(L.)amazonensis* is characterised as a disease that is difficult to control and requires specific measures depending on the area of occurrence. Therefore, besides the establishment of early diagnosis and treatment, the proper identification of the species of*Leishmania* and the determination of its area of distribution are essential to the planning and adoption of prevention measures and the reduction of the exposure of the human population to the vector (Dorval et al. 2006, MS/SVE 2010).

Considering that *L*. *(L.)infantum* and*L*. *(L.)amazonensis* were identified in Corumbá and the lack of studies on aetiology of human and canine cases of leishmaniases, both visceral and cutaneous forms, in addition to clinical and epidemiological aspects, attention special should be given to the identification by genotyping of the parasites.

Experimental infection studies, undertaken by this research group, with both species, are currently underway and are necessary to gain a better understanding of the parasite-vector interaction. Studies for the identification of reservoir hosts should also be conducted, since some wild and domesticated mammals are considered to be hosts of different species of *Leishmania* (Ashford 2000).

In short, *L. (L.) amazonensis* DNA was detected for the first time in*Lu. cruzi* collected in the urban area of Corumbá, an endemic area for VL and CL. The authors would like to emphasize the importance of this finding, since *Lu*. *cruzi*, a suspected vector of*L*. *(L.)infantum* and adapted to the urban environment, could contribute to the dispersion and urbanisation of*L*. *(L.)amazonensis*. It is further relevant the fact that this species has been found associated with human and canine VL cases. Therefore, these findings point to the need for further investigation into the possible role of this sandfly as vector of this parasite.
